# Raman Microscopic Analysis of Internal Stress in Boron-Doped Diamond

**DOI:** 10.3390/ma8052782

**Published:** 2015-05-22

**Authors:** Kevin E. Bennet, Kendall H. Lee, Jonathan R. Tomshine, Emma M. Sundin, James N. Kruchowski, William G. Durrer, Bianca M. Manciu, Abbas Kouzani, Felicia S. Manciu

**Affiliations:** 1 Division of Engineering, Mayo Clinic, Rochester, MN 55905, USA; E-Mails: Bennet.Kevin@mayo.edu (K.E.B.); Kruchowski.James@mayo.edu (J.N.K.); 2 Department of Neurologic Surgery, Mayo Clinic, Rochester, MN 55905, USA; E-Mails: Lee.Kendall@mayo.edu (K.H.L.); Tomshine.Jonathan@mayo.edu (J.R.T.); 3 Department of Physics, University of Texas at El Paso, El Paso, TX 79968, USA; E-Mails: emsundin@miners.utep.edu (E.M.S.); wdurrer@utep.edu (W.G.D.); bianca.manciu@gmail.com (B.M.M.); 4 School of Engineering, Deakin University, Waurn Ponds, Victoria 3216, Australia; E-Mail: abbas.kouzani@deakin.edu.au

**Keywords:** confocal Raman mapping, induced stress, boron-doped diamond

## Abstract

Analysis of the induced stress on undoped and boron-doped diamond (BDD) thin films by confocal Raman microscopy is performed in this study to investigate its correlation with sample chemical composition and the substrate used during fabrication. Knowledge of this nature is very important to the issue of long-term stability of BDD coated neurosurgical electrodes that will be used in fast-scan cyclic voltammetry, as potential occurrence of film delaminations and dislocations during their surgical implantation can have unwanted consequences for the reliability of BDD-based biosensing electrodes. To achieve a more uniform deposition of the films on cylindrically-shaped tungsten rods, substrate rotation was employed in a custom-built chemical vapor deposition reactor. In addition to visibly preferential boron incorporation into the diamond lattice and columnar growth, the results also reveal a direct correlation between regions of pure diamond and enhanced stress. Definite stress release throughout entire film thicknesses was found in the current Raman mapping images for higher amounts of boron addition. There is also a possible contribution to the high values of compressive stress from sp^2^ type carbon impurities, besides that of the expected lattice mismatch between film and substrate.

## 1. Introduction

Boron-doped diamond (BDD) and other diamond-based materials have been subjects of interest in the past years, especially for their potential in electrochemistry and voltammetry applications [1,2,3,4,5,6,7,8,9]. In addition to overall material quality, of concern when considering the use of electrodes fabricated from BDD coated tungsten rods in neurosurgical implantations into the brain is the unwanted occurrence of film delamination and dislocation. Deep brain stimulation (DBS), which is the planned application of the current work, is a currently Food and Drug Administration (FDA) approved technique employed for treatment of patients with movement disorders such as Parkinson’s disease. By coupling DBS with fast-scan cyclic voltammetry (FSCV) technique, for the purpose of biochemical sensing and feedback control of stimulation parameters, the release of neurotransmitters in the brain can be monitored.

Besides the inherent inhomogeneity of any sample, a significant factor affecting the relevant properties of such thin films and, consequently, long-term electrode performance, is the induced stress during or after deposition. Part of this stress could be reduced by choosing an appropriate substrate for diamond deposition. This would entail matching substrate parameters such as surface energy, lattice, structure, and coefficient of thermal expansion with those of diamond. While there is no ideal candidate as a substrate for diamond, tungsten is the element that most closely approaches diamond in terms of surface energy (e.g., at melting temperature the surface energy of tungsten is approximately 3111 ergs/cm^2^ and that of diamond is about 3300 ergs/cm^2^) and lattice constant (e.g., tungsten’s lattice constant is 3.16 Å and diamond’s is 3.57 Å, with about 10% lattice mismatch) [10,11,12,13]. On the other hand, tungsten has a significantly different structure (*i.e.*, body-centered cubic for tungsten and face-centered cubic for diamond) and a greater coefficient of thermal expansion (*i.e.*, 4.3 × 10^−6^/K for tungsten and 1.18 × 10^−6^/K for diamond) [10,12]. In this context, the reported increase of about 10%–15% in thermal expansion for doped synthetic diamond over that of undoped diamond ([11,12,13] and references therein) is expected to positively impact the characteristics of heavily doped BDD films, not only by increasing the desired electrical conductivity, but also by reducing the induced stress.

Other contributing factors that affect the residual stress induced in the grown material and its other properties are the shape of the substrate, the growth temperature, and the feed gas pressures employed during fabrication [7,8,9,10,11,12,13,14,15,16,17,18,19,20,21]. Previous studies on synthetic diamond showed that the residual stress has two main components: The intrinsic stress and the extrinsic stress [14,15,16,17,18]. Defects within the film (e.g., nondiamond carbon phases) and lattice mismatch between the deposited material and the substrate are the main reasons for intrinsic stress. Extrinsic stress is likely to happen because of the difference between the thermal expansion coefficients of the diamond and substrate used. The latter type of stress, also known as thermal stress, is mainly compressive. Both tensile and compressive stresses were observed in polycrystalline diamond and BDD films grown by chemical vapor deposition (CVD) [19,20]. Partial release of the stress has been demonstrated for undoped, highly crystalline diamond thin films under proper tuning of growth temperature and amounts of feed gases [18]. More complex, interrelated aspects should be considered for stress release in the case of BDD polycrystalline films. Besides boron incorporation, which results in expansion of the diamond lattice [19], grain boundaries, which are the locations of impurity accumulations, could add to stress relaxation due to the elastic strain induced in the grains [16]. Based on confocal Raman mapping analysis, a comparative assessment of fabrication parameters (*i.e.*, amount of feed gases, shape, type, and temperature of the substrate, which are described later in Section 3.1.) and their contributions to the observed induced stress for doped and undoped diamond films grown on tungsten rods is presented and discussed in this work.

Although other techniques have been employed in analyses of residual stress such as X-ray diffraction [20,21] and the curvature technique [16], Raman spectroscopy still remains the most used method for this type of characterization. One reason is that the Raman technique provides knowledge of the composition of the material at a molecular level, as well as an evaluation of internal stress through the diamond Raman peak shifts. While the majority of previous Raman studies of BDD films have been based on spectral analysis, the confocal Raman mapping used in the current investigations allows for qualitative and relative quantitative evaluations of the material under study through direct visualization of the local distribution of film constituents (e.g., pure diamond, boron incorporation and accumulation, and non-diamond, carbon sp^2^ impurities). Additionally, the Raman stress mapping images provide an assessment of stress location (*i.e.*, close to the substrate or to the surface) and its progression throughout the film thickness. This information is essential for improving the properties of BDD coated electrodes, and, consequently, their reliability for DBS studies. The results of this work represent an important component of understanding the interdependence of physical and chemical properties of BDD films necessary for development of stable and high quality electrodes.

## 2. Results and Discussion

**Figure 1:**
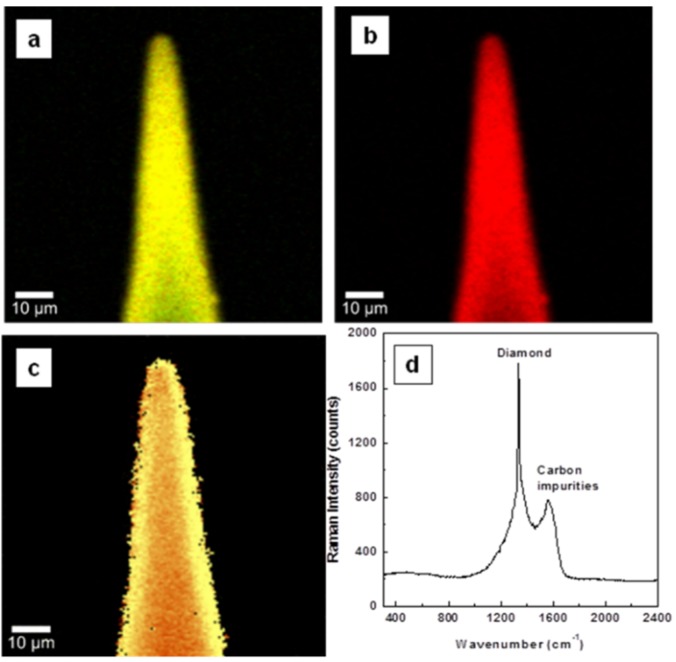
Surface confocal Raman images of undoped diamond film mapping the spatial distribution of: (**a**) diamond and sp^2^ carbon, (**b**) diamond only, and (**c**) induced stress. Red pseudo-color is used for diamond and green for sp^2^ carbon impurities. While in image (**a**) yellow originates from a combination of red and green, in the stress image (**c**), bright yellow and brown correspond to higher or lower induced stress, respectively. (**d**) integrated Raman spectrum of image (**a**).

**Figure 2:**
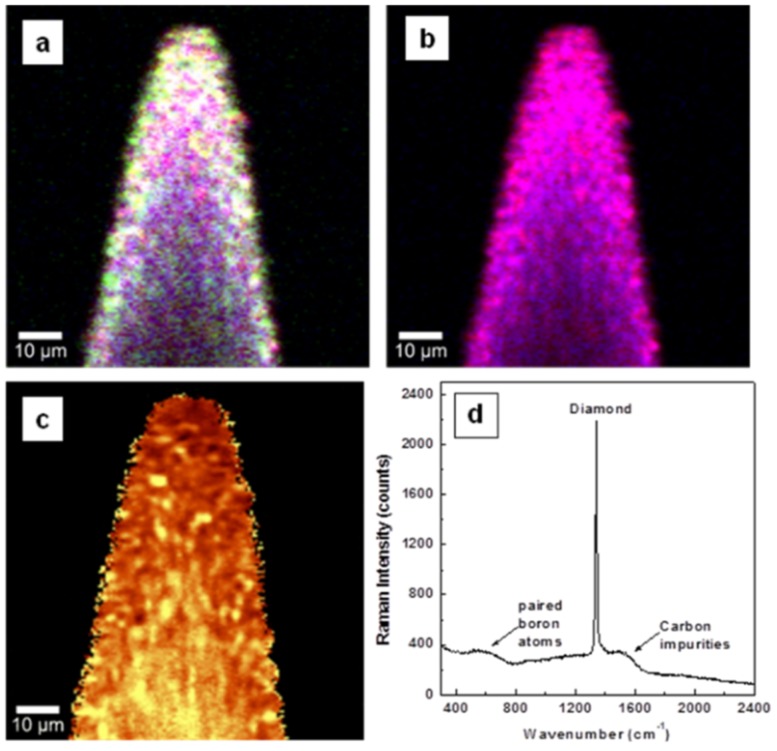
Surface confocal Raman images of lightly doped diamond film mapping the spatial distribution of: (**a**) diamond (red), boron incorporation (blue), and sp^2^ carbon impurities (green), (**b**) diamond (red) and boron incorporation (blue), and (**c**) induced stress. Yellow pseudo-color in the stress image corresponds to higher induced stress. White, magenta, and turquoise colors observed in images (**a**) and (**b**) are due to combinations of red with blue and green, of red with blue, and of blue with green, respectively. (**d**) integrated Raman spectrum of image (**a**).

**Figure 3:**
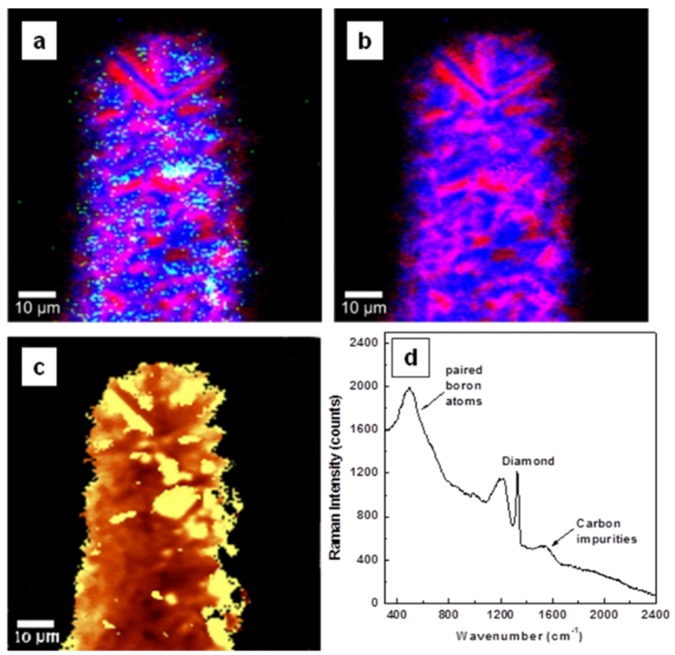
Surface confocal Raman images of heavily doped diamond film mapping the spatial distribution of: (**a**) diamond (red), boron incorporation (blue), and sp^2^ carbon impurities (green), (**b**) diamond (red) and boron incorporation (blue), and (**c**) induced stress. Yellow pseudo-color in the stress image corresponds to higher induced stress. White, magenta, and turquoise colors observed in images (**a**) and (**b**) are due to combinations of red with blue and green, of red with blue, and of blue with green, respectively. (**d**) Integrated Raman spectrum of image (**a**).

Investigations by confocal Raman mapping of material morphology and internal stress in the currently grown samples for undoped, lightly boron-doped, and heavily boron-doped diamond thin films, are presented in Figure 1a–d, Figure 2a–d, and Figure 3a–d, respectively. The morphological measurements were performed by selecting characteristic Raman vibrational lines at 1332 ± 2 cm^−1^ for diamond, around 1200 cm^−1^ and 500 cm^−1^ for boron incorporation and accumulation in the diamond lattice (*i.e.*, paired boron atoms), respectively, and the band centered at 1500 cm^−1^ corresponding to sp^2^ carbon impurities [1,4,8,22,23,24]. For a relative quantitative identification of these constituents, three pseudo-colors (*i.e.*, red for diamond, blue for BDD, and green for carbon impurities) were used. Comparison of the results of Figure 1a with those of Figure 2a and Figure 3a reveals a decrease of sp^2^ carbon (green) with addition of boron. This observation is confirmed in Figure 1d, Figure 2d, and Figure 3d, where the integrated Raman spectra of these images are presented. A decrease in the intensity of the feature around 1500 cm^−1^ can be seen in Figure 2d and Figure 3d as compared with the one in Figure 1d. Thus, boron addition not only contributes to the conductivity of the films [9], but also promotes higher quality of the deposited material, by reducing the amount of unwanted carbon impurities and promoting a more rapid crystallization.

For easier examination of film uniformity as related to boron incorporation, the visualization of sp^2^ carbon is excluded in Figure 1b, Figure 2b, and Figure 3b. Besides the expected color trend from red (*i.e.*, Figure 1b of undoped material) to blue for a higher boron doping level, in both Raman mapping images presented in Figure 2b and Figure 3b the signature of pure diamond crystallites (red color) is still visible; a phenomenon originating from the known preferential incorporation of boron into the diamond lattice [5,6,7,8,22,23,24].

There is also slightly more uniformity in the incorporation of boron for a lower amount of doping (*i.e.*, dominant magenta color, which is a combination of red and blue). This remark can be explained by the tendency of boron to first substitute for carbon, with the outcome of a magenta pseudo-color, then to incorporate interstitially or even to aggregate for higher doping amounts, resulting in a blue pseudo-color. In this respect, it is worth pointing out that the boron incorporation mechanism for concentrations that impart metallic-like or even superconductive behavior to naturally non-conductive diamond (*i.e.*, more than 10^20^ atoms/cm^3^) is still not completely established [11]. Some of the reasons for these uncertainties are the difficulty of accurately determining boron concentration and the low magnitudes of the diamond lattice parameters and thermal expansions. While the tetrahedral arrangement of carbon in the diamond structure is directly related to its low lattice expansion parameter, it is this arrangement that makes diamond such a remarkable material from the points of view of both mechanical and thermal conductive properties, and that makes it a good prospect for future electronic applications. The aggregation of interstitial boron atoms [8,22,23,24] and the formation of isolated, interacting boron pairs, which is observable in the current Raman spectra by the presence of the band around 500 cm^−1^, were reported to be the main causes contributing to the dramatic increase of the thermal expansion coefficient of diamond (by several, or dozens, of times larger than that of pure diamond) in heavily doped BDD films [11].

The evaluation of the induced stress in the samples is shown in Figure 1c, Figure 2c, and Figure 3c. Surface confocal Raman mapping was performed to acquire these images, which account for the shift of the characteristic optical-phonon vibration of diamond at 1332 cm^−1^. More of the bright yellow pseudo-color corresponds to more induced stress. The *Advanced Fitting Tool* of the *WITec Project Plus* software was employed to fit each spectrum of the entire Raman spectral data set with a Lorentzian function (*i.e.*, at every image pixel a Raman spectrum is recorded). It has been shown that this vibrational line shifts around 3 cm^−1^/1 GPa when the material is under stress [19,25,26,27]. Also, a peak shift to higher or lower frequency was associated with compressive or tensile stress, respectively [25].

A compressive stress is found, with 4.5 ± 2 to 12 ± 2 cm^−1^ (*i.e.*, 1.5 to 4 GPa) positive shifts and with higher values for the undoped samples than for the doped ones. This statement is supported by the overall lighter, towards-yellow pseudo-color seen in the image presented in Figure 1c and in parts of Figure 2c and Figure 3c. Different causes can contribute to this behavior. For example, a closer, comparative look at these images regarding only the presence of pure diamond crystallites (red pseudo-color in Figure 2b and Figure 3b) demonstrates that more stress (yellow pseudo-color) directly associates with them. This effect is obvious for the heavily doped sample (see Figure 3b,c), as boron addition promotes faster growing rates and formation of larger crystallites. Complementarily, if the decrease in stress is sought, good visual agreement is evident between regions of brown false color and the ones corresponding to higher amounts of boron doping (*i.e.*, blue regions). Thus, as discussed above, the main argument for the stress release could be based on the strong increase in the thermal expansion of diamond with boron addition. Due to carbon impurities, there might be some additional induced surface stress, which is most visible in Figure 1c and in some parts of Figure 3c. The contribution of such impurities to the surface stress in the case of heavily doped samples can be explained by the known phenomenon of their accumulation at the boundaries of BDD crystallites [8,20,21,22,23]. The presence of only nanocrystallites in the undoped sample is thus also likely to play a role in the formation of the relatively high amount of sp^2^ carbon observed in this case. The broadness seen in the integrated spectrum presented in Figure 1d at the base of the Raman vibrational line characteristic of diamond supports this affirmation.

**Figure 4:**
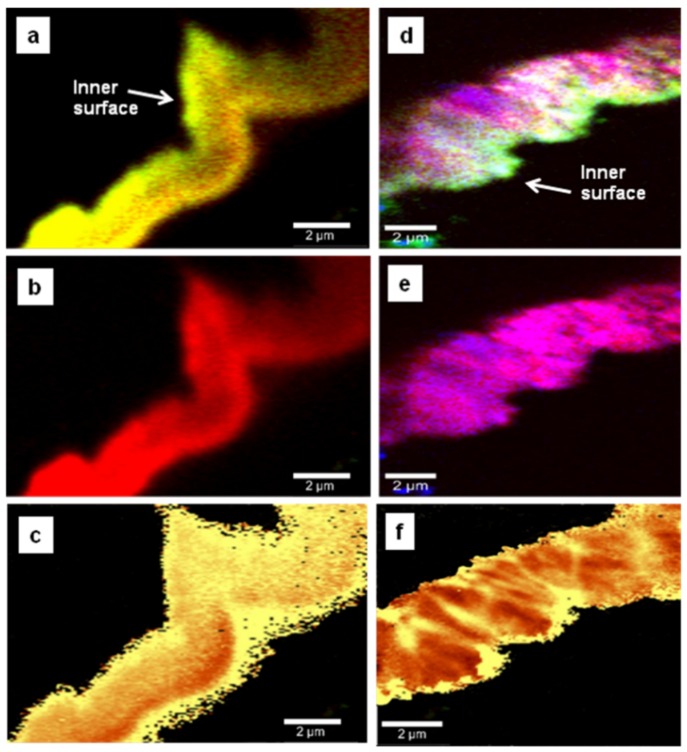
Side-wall confocal Raman images of undoped diamond thin film mapping the spatial distribution of (**a**) diamond (red) and sp^2^ carbon (green), (**b**) diamond only, and (**c**) induced stress. Side-wall confocal Raman images of lightly boron-doped thin film showing: (**d**) all the material constituents, (**e**) diamond and boron incorporation, and (**f**) induced stress. Red, blue, and green pseudo-colors are used in image (**d**) for diamond, boron, and carbon impurities, respectively. While in image (**a**) yellow originates from superposition of red and green, in the stress images (**c**) and (**f**), bright yellow and brown correspond to higher or lower induced stress, respectively.

**Figure 5:**
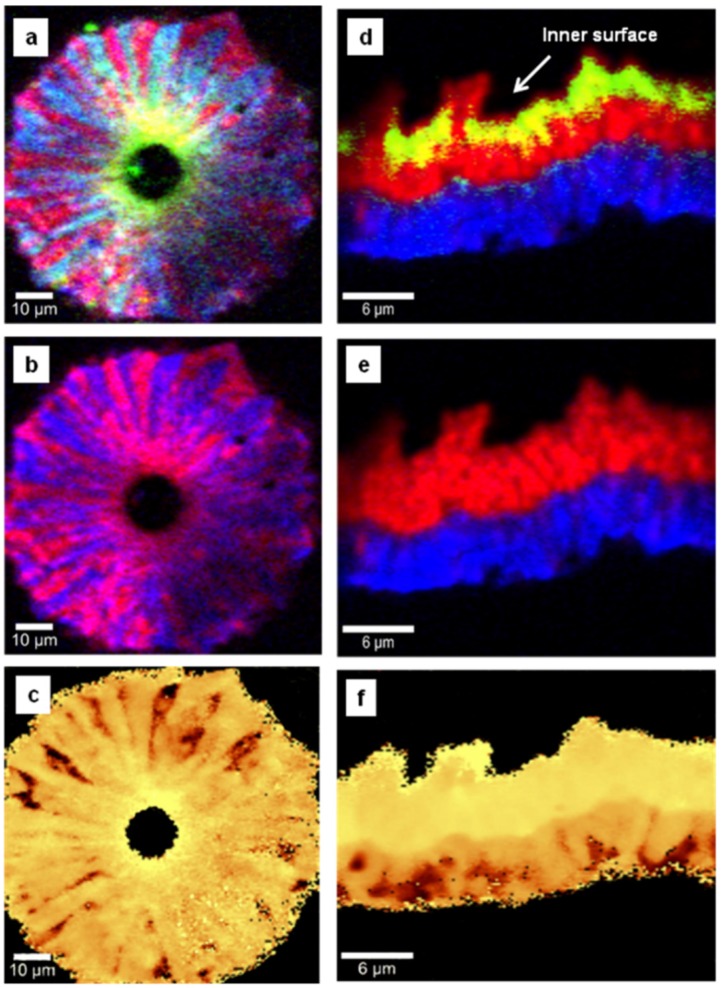
Side-wall confocal Raman mapping images of: (**a**)–(**c**) a heavily boron-doped film and its corresponding stress mapping analysis, and (**d**)–(**f**) two consecutive layers grown without and with boron addition, together with the associated stress map. Red, blue, and green pseudo-colors are used for diamond, boron, and carbon impurities, respectively. Yellow pseudo-color in the stress images corresponds to higher induced stress.

Another important source of the induced stress in the material is the lattice mismatch between tungsten and diamond, which should occur more near the interface between the two materials. To asses this interface effect, we performed side-wall confocal Raman mapping, and the results are presented in Figure 4a–f and Figure 5a–f. Even though no evidence of tungsten carbide formation was found, Figure 4a,d and Figure 5a,d show a strong accumulation of carbon impurities close to the surface of the tungsten rods. Again, a lower amount of trimethylborane (TMB) feed gas used during deposition results in a more uniform incorporation of boron throughout the entire film thickness (a relatively uniform magenta color is observed in Figure 4d). For a considerably higher amount of TMB, preferential boron incorporation, with an obvious radial, columnar growth, is occurring (see Figure 5b). Comparison between the images for chemical composition of the films and the induced stress (see Figure 4a,c,d,f and Figure 5a,c) reveals an obvious correlation between the stress and the excessive presence of carbon impurities at the interface with tungsten. Shifts of the diamond vibrational line with values as high as 20 ± 1 cm^−1^ (6.7 GPa) were obtained for these bright yellow regions. We do not exclude the possibility that the lattice mismatch has some contribution to such high values of compressive stress.

There is also an indication of a radial, channeled release of the stress towards the film surface, where much smaller stress values were found (between 1.5 and 4 GPa). Thus, the relaxation of the grain boundaries at the film surface could also have an influence. As the growth favors the directions with lower strain energy density, a clear analogy between the regions where the boron presence is more visible (blue pseudo-color) and the ones with less stress (dark brown pseudo-color) is observed, especially in Figure 5b,c. No evidence of defects or dislocations that would degrade material quality or create other types of internal stresses is detected.

In order to further investigate our previous assumption that the origin of the stress release is mainly due to enhancement of the thermal expansion parameter of diamond with boron incorporation, we performed side-wall confocal Raman analysis of a two-layer BDD thin film with each layer grown under the same conditions but with different amounts of TMB. These images, which are presented in Figure 5d–f, besides proving the capability of confocal Raman mapping in detecting differences in doping, also give new insights into this matter and its relation to the growth process. For instance, the location of the strongest presence of carbon impurities at the interface with tungsten (see Figure 5d) is different from that of the most intense observed stress (see Figure 5f). This observation implies that the mismatch between the diamond and the substrate is still the dominant factor in the induced stress. More importantly, the noticeable color change corresponding to the induced stress in the two thin films, combined with almost no evidence of carbon impurity accumulation at the interface between them, validates the importance of boron doping for stress release. The nearly uniform distribution of the stress in the entire volume of the undoped diamond films (*i.e.*, at their surfaces and across their thicknesses, as seen in Figure 1c, Figure 4c and Figure 5f, respectively) can be understood in terms of an effective increase in the number of atoms at the surface for the reduced dimensions of the nanocrystallites and absence of a preferential growth direction. The high number of atoms at the surface is likely to affect the grain boundary relaxation effect, while the temperature at the beginning of the growth process will influence the presence of sp^2^ carbon, as it might not be high enough to provide sufficient energy for diamond crystallization. Indeed, stabilization of crystal formation is visible in Figure 5d,e for thicker films in comparison with the scenario revealed in Figure 4a,b.

## 3. Experimental Section

### 3.1. Fabrication of Boron-Doped Diamond Thin Films

The BDD films were grown in a hot filament chemical vapor deposition (CVD) reactor using a mixture of CH_4_/H_2_ gases (nominally 99% H_2_ and 1% CH_4_) at a total chamber pressure of 20 Torr. The current samples were grown on tungsten rods that were initially electrochemically etched by immersion in 1 M NaOH, then abraded by sonication for 30 min in diamond powder (100 nm, Engis, 105 West Hintz Road, Wheeling, IL, USA)/isopropyl alcohol grit slurry, and finally cleaned by rinsing in deionized water. 

For more uniform deposition of the BDD films on the cylindrical shape of the electrodes, the substrate rotation mode of this custom-built reactor (at the Mayo Clinic) was employed. The tungsten rods were mounted parallel to the central axis of the filament coil. A Proportional Integral Derivative (PID) software-based control loop allowing the power to be ramped up to 450 W was used for achieving temperatures of 2300 °C for the filament and about 800 °C for the substrate. The stabilities of these two different temperatures were monitored during film deposition with a Spectrodyne DFP 2000 optical pyrometer and an Omega Engineering type K thermocouple, respectively. Two different amounts of trimethylborane (TMB) flow gas (1000 ppm in hydrogen, Voltaix Products, Branchburg, NJ, USA), namely, chamber concentrations of 10 ppm (2 sccm TMB/hydrogen, 2 sccm methane, and 196 sccm hydrogen) and 100 ppm (20 sccm TMB/hydrogen, 2 sccm methane, and 178 sccm hydrogen), were released into the chamber for obtaining lightly doped and heavily doped thin films, respectively. Undoped samples were also prepared without addition of TMB, under identical temperature growth conditions and using 2 sccm methane and 198 sccm hydrogen.

### 3.2. Raman System

The confocal Raman measurements were acquired at ambient conditions, in a backscattering geometry, with an *alpha 300R WITec* system (WITec GmbH, Ulm, Germany) using the 532 nm excitation of a frequency-doubled Nd:YAG laser. While 100X/numerical aperture (NA) of 0.90 and 50X/NA of 0.75 objective lenses were used for side-wall measurements of BDD films, to reduce the optical effects of the inherent curvature of the tungsten rods, a 20X/NA of 0.40 objective lens was used for acquiring such data on the surfaces of the films. An array of 150 × 150 Raman spectra were recorded for all Raman images with an integration time of 50 ms/spectrum. All the surface Raman mapping images were acquired with 85 µm × 85 µm scan sizes. Appropriate scan size dimensions, dependent on film thickness, were considered for side-wall maps, while maintaining a similar scale-bar in these images. Also, for these measurements, the samples were cross-sectionally cleaved along their diameters.

Although the spot size of the laser, and implicitly the optical resolution, is diffraction limited, the image quality was improved by using an oversampling procedure. This procedure, applicable for more than 20,000 pixels per image, consists of using increments smaller than the lateral spectral resolution, which varies between 255 and 575 for the currently used excitation of 532 nm and different objective lenses. On the other hand, in confocal Raman microscopy, only a relative determination of concentrations is possible for multi-constituent solid sample materials, as the pseudo-color of a pixel is graded relative to the intensity of the constituent detected at that location (in every pixel a Raman spectrum is recorded).

Besides the *WITec Control* software, which controls the piezoelectric stage for sample scanning and is normally employed for data acquisition, the *Advanced Fitting Tool* of the *WITec Project Plus* software was used for stress data analysis. In this context, small shifts can be detected, as fitting the peaks allows the maxima to be determined with a precision better than 1 cm^−1^, although the sampling resolution can be lower.

## 4. Conclusions

In this study we present a detailed analysis by confocal Raman microscopy of the induced stress on a series of undoped and boron-doped diamond thin films grown by chemical vapor deposition on tungsten rods. For consistent film deposition on the cylindrical shapes of these substrates, the custom-built reactor’s capability of tungsten rod rotation was employed.

The current results demonstrate a relatively uniform presence of the stress throughout the entire pure diamond film and its radial release that becomes dependent on the sample chemical composition with boron addition. An obviously stronger stress release for a higher amount of boron doping is revealed by some of the surface Raman mapping images (see, and compare, Figure 1c, Figure 2c, and Figure 3c), especially by the side-wall image presented in Figure 5f, where two consecutively grown layers, without and with boron doping, are mapped. Besides an anticipated enhanced compressive stress observed close to the interface between the film and the tungsten substrate, which is due mainly to the lattice mismatch between the two materials, there is also a potential contribution from sp^2^ carbon impurities to the high compressive stress values obtained. The high correlation of the presence of pure diamond with the strongest intensity of induced stress, together with the observed stress release with doping, make us conclude that boron addition not only contributes to the electrical conductivity of the material necessary for biosensing applications, but also to its improvement towards desired overall quality. Unwanted occurrence of film delaminations and dislocations during neurosurgical implantation of BDD-based electrodes can negatively affect their reliability in biosensing.
